# T1 mapping as a quantitative imaging biomarker for diagnosing cervical cancer: a comparison with diffusion kurtosis imaging

**DOI:** 10.1186/s12880-024-01191-x

**Published:** 2024-01-10

**Authors:** Zanxia Zhang, Jie Liu, Yong Zhang, Feifei Qu, Robert Grimm, Jingliang Cheng, Weijian Wang, Jinxia Zhu, Shujian Li

**Affiliations:** 1 Department of MRI, The First Affiliated Hospital of Zhengzhou University, No. 1 Jianshe Dong Road, 450052 Zhengzhou, Henan China; 2 MR Collaboration, Siemens Healthcare Ltd, Beijing, China; 3 MR Application, Siemens Healthcare GmbH, Predevelopment, Erlangen, Germany

**Keywords:** Cervical cancer, T1 mapping, Magnetic resonance imaging, Diffusion kurtosis imaging

## Abstract

**Background:**

T1 mapping can potentially quantitatively assess the intrinsic properties of tumors. This study was conducted to explore the ability of T1 mapping in distinguishing cervical cancer type, grade, and stage and compare the diagnostic performance of T1 mapping with diffusion kurtosis imaging (DKI).

**Methods:**

One hundred fifty-seven patients with pathologically confirmed cervical cancer were enrolled in this prospectively study. T1 mapping and DKI were performed. The native T1, difference between native and postcontrast T1 (T1diff), mean kurtosis (MK), mean diffusivity (MD), and apparent diffusion coefficient (ADC) were calculated. Cervical squamous cell carcinoma (CSCC) and adenocarcinoma (CAC), low- and high-grade carcinomas, and early- and advanced-stage groups were compared using area under the receiver operating characteristic (AUROC) curves.

**Results:**

The native T1 and MK were higher, and the MD and ADC were lower for CSCC than for CAC (all *p* < 0.05). Compared with low-grade CSCC, high-grade CSCC had decreased T1_diff_, MD, ADC, and increased MK (*p* < 0.05). Compared with low-grade CAC, high-grade CAC had decreased T1_diff_ and increased MK (*p* < 0.05). Native T1 was significantly higher in the advanced-stage group than in the early-stage group (*p* < 0.05). The AUROC curves of native T1, MK, ADC and MD were 0,772, 0.731, 0.715, and 0.627, respectively, for distinguishing CSCC from CAC. The AUROC values were 0.762 between high- and low-grade CSCC and 0.835 between high- and low-grade CAC, with T1_diff_ and MK showing the best discriminative values, respectively. For distinguishing between advanced-stage and early-stage cervical cancer, only the AUROC of native T1 was statistically significant (AUROC = 0.651, *p* = 0.002).

**Conclusions:**

Compared with DKI-derived parameters, native T1 exhibits better efficacy for identifying cervical cancer subtype and stage, and T1_diff_ exhibits comparable discriminative value for cervical cancer grade.

## Background

Cervical cancer is a common malignancy of the female reproductive tract and ranks second among the causes of cancer-related deaths among women in developing countries [1]. Cervical cancer is typically preoperatively diagnosed based on histopathological biopsies. However, cervical cancer biopsies reflecting the focal lesions instead of the whole tumor may cause errors. Therefore, imaging studies could play a vital role in evaluating cervical cancer [2]. Magnetic resonance imaging (MRI) of the cervix, which consists of T2-weighted imaging (T2W), diffusion-weighted imaging (DWI), and dynamic contrast material-enhanced MRI, is the most commonly used imaging modality for preoperatively evaluating cervical cancer. MRI is recommended in the 2018 International Federation of Gynecology and Obstetrics (FIGO) guidelines [3, 4].

Conventional DWI and apparent diffusion coefficient (ADC) measurements assume Gaussian behavior for water diffusion and use a simple monoexponential fit of the signal decay data [5]. However, microstructural barriers, including both cellular density and cellular membranes, obstruct water diffusion. Diffusion kurtosis imaging (DKI) is an advanced DWI technique based on a non-Gaussian model that can reveal pathological features with respect to cell density and microstructural complexity, which is superior to the monoexponential model [6]. Previous studies concerning DKI have been conducted on patients with cervical cancer and have shown the potential value of DKI in diagnosing and characterizing cervical cancer [7–10]. However, DKI is not widely used in clinical practice owing to its long scan time and complex post-processing.

T1 mapping is a rapid, noninvasive MR technique that permits quantitatively analyzing biological tissue characteristics by measuring the longitudinal relaxation time (T1 value) in image voxels [11]. T1 mapping is characterized by the absence of complex mathematical models during the MR scan and by simple image post-processing, making it easier to use in clinical practice. This quantitative technique has been applied to studies on myocardial lesions, liver fibrosis, renal tumors, cervical cancer, and chronic pancreatitis [12–17]. E.g.,Wang W et al. used extracellular volume (ECV) fraction based on T1 mapping for preoperative identifying of lymphovascular space invasion (LVSI) in 79 cervical cancer patients as compared to dynamic contrast-enhanced (DCE) MRI, finding better performance of T1mapping than DCE for differentiating LVSI [17]. As we previously reported, T1 mapping has shown high value in predicting prognostic pathologic factors and risk factors for recurrence in cervical cancer [18–20]. However, most of these studies have focused on cervical squamous cell cancer [18, 19], and few systematic studies have investigated the potential clinical applications of T1 mapping in the characterization of cervical adenocarcinoma [16]. Additionlly, relevant studies that have assessed the histological characteristics of cervical cancer using T1 mapping and DKI are still in the early stages [20]. Therefore, this study was conducted to assess the performance of T1 mapping in distinguishing between cervical cancer type, grade, and stage and to compare T1 mapping with diffusion kurtosis imaging (DKI).

## Materials and methods

### Study participants

**Fig. 1:**
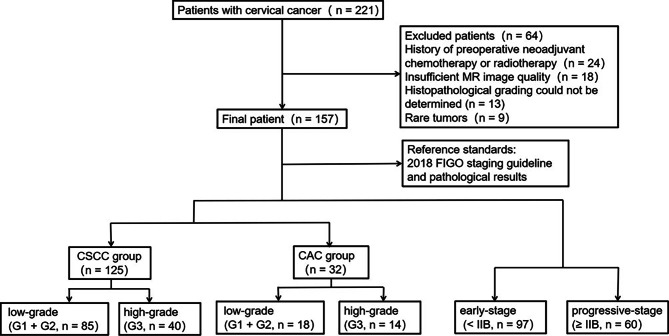
Flowchart of patient population

This was a prospectively single-center study approved by the Institutional Review Board of our hospital. All study participants provided written informed consent. Between October 2018 and November 2021, 221 consecutive women with suspected cervical cancer underwent pelvic MRI, including T1 mapping and DKI, before surgery at our institution. The inclusion criteria were (1) no contraindications to MRI examination or pelvic metal artifacts; (2) no surgery, chemotherapy, or radiotherapy before MRI examination; and (3) cervical cancer confirmed by biopsy or surgical pathology after MRI examination. The exclusion criteria were (1) images not meeting analysis requirements, such as incomplete study-related sequences, severe motion artifacts or metallic artifacts; (2) history of receiving chemotherapy or radiation therapy prior to MRI examination; 3)incomplete pathological findings, such as undetermined histological subtype or pathological grade by pathological examination; and 4) rare tumors, such as adenosquamous carcinoma of the cervix and neuroendocrine tumors. Finally, 157 patients with cervical cancer were enrolled, of whom, 102 had their diagnosis confirmed by surgery and 55 by biopsy. The mean interval between MRI and surgery or biopsy was 8.5 days (range, 2–15 days). A pathologist (W.N., an attending physician) with 9 years of experience diagnosing uterine malignancies and who was blinded to the original diagnosis re-evaluated the histopathology slides from all participants. Participants were divided into the cervical squamous cell carcinoma (CSCC), adenocarcinoma (CAC), early-stage (< IIB), or advanced-stage (≥ IIB) groups based on the 2018 FIGO staging guidelines and histopathological findings (4). Participants in the CSCC and CAC groups were further divided into high-grade (G3) and low-grade (G1 + G2) groups (Fig. 1).

### Image acquisition

The pelvic MRI examinations were performed using a3T MR scanner (MAGNETOM Skyra; Siemens Healthcare, Erlangen, Germany) with an 18-channel phased-array body coil. Patients were instructed to fast for 4 to 6 h before the MR examination. Multiparametric MRI protocols were performed on all patients. First, axial and sagittal turbo spin-echo T2-weighted imaging (T2WI), axial T1-weighted imaging (T1WI), axial DWI, axial T1 mapping and axial DKI were performed. Next, gadolinium diethylenetriamine pentaacetic acid (Gd-DTPA; Magnevist, Schering, Berlin, Germany) was administered intravenously at 1.5 mL/s (total dose, 0.1 mmol per kg of body weight) using a power injector, followed by a 20-mL saline flush. Axial postcontrast T1 mapping sequence scans were obtained 5 min after contrast injection. Finally, a volumetric interpolated breath-hold examination (VIBE)-T1W sequence was acquired after enhancement. T1 maps were acquired via the T1 mapping sequence (3D VIBE-based sequence) using the B1-corrected variable flip angle (VFA). A B1 map sequence was used for matching before VFA sequence acquisition and for automatic correction afterward. DKI scans were acquired using a readout-segmented echo-planar sequence (RESOLVE) [21]. Native and postcontrast T1 maps with B1 correction were generated in line after data acquisition by the MapIt software (Siemens Healthcare, Erlangen, Germany) using the method described by Deoni et al. [22]. The total duration of an imaging session was 46 min 57s. Table 1 lists the protocol details.

**Table 1:** MRI sequence parameters

Parameters	T1WI	T2WI	DKI	T1 mapping (pre-/post-contrast-enhanced)	B1 map for T1 mapping	Pre-/Post-contrast T1WI
Imaging technique Orientation	TSE Axial	TSE Axial	RESOLVE Axial	3D VIBE Axial	Turbo-Flash Axial	3D VIBE Axial
TR (ms)	450	3000	4160	5.01	8980	6.16
TE (ms)	18	116	51	2.3	1.83	3
Field of view (mm^2^)	320 × 320	180 × 180	320 × 240	380 × 304	380 × 309	320 × 320
Slice thickness (mm)	4	4	4	4	8	2
No. of slices	25	25	25	32	32	60
Acquisition matrix	448 × 314	384 × 288	192 × 144	224 × 168	64 × 52	320 × 240
Voxel size (mm^3^)	0.7 × 0.7 × 4.0	0.7 × 0.5 × 4.0	1.7 × 1.7 × 4.0	0.8 × 0.8 × 4.0	6.0 × 6.0 × 8.0	1.0 × 1.0 × 2.0
b-values (s/mm^2^)	NA	NA	0, 500, 1000, 1500, 2000	NA	NA	NA
No. of directions	NA	NA	3	NA	8	NA
Flip angle (degrees)	180	160	180	3, 5, 8, 10, 13, 15		9
Bandwidth (Hz/pixel)	254	200	1002	300		450
Acquisition time	1 min 33 s	3 min 20 s	9 min 38 s	30 s		56 s

Abbreviations: T1WI, T1-weighted imaging; T2WI, T2-weighted imaging; DWI, diffusion-weighted imaging; TSE, turbo spin-echo; RESOLVE, readout-segmented echo-planar sequence; VIBE, volumetric interpolated breath-hold examination; TR, repetition time; TE, echo time; NA, not applicable

### Image analysis and measurements

The acquired data were transferred to a post-processing workstation using Syngo.via software (Syngo.via, Siemens Healthcare). DKI was processed using a prototype software (MR Body Diffusion Toolbox, Siemens Healthcare, Erlangen, Germany) in the Frontier post-processing platform to obtain mean kurtosis (MK), mean diffusivity (MD), and apparent diffusion coefficient (ADC) maps using the method described by Jensen et al. [23]. The mathematical models of DKI with five b-values (0, 500, 1000, 1500, and 2000 s/mm2) was based on the equation:

$$ \frac{{{S_b}}}{{{S_0}}} = exp\left( { - b \cdot {D_{app}} + \frac{1}{6}{b^2} \cdot {D_{app}} \cdot {K_{app}}} \right)$$,

where ${\text{S}}_{\text{b}}$ and S0 are the signal intensity of diffusion weighting b and without the diffusion gradient applied, respectively, ${\text{D}}_{\text{a}\text{p}\text{p}}$ is the corrected apparent diffusion coefficient derived from the non-Gaussian model, and ${\text{K}}_{\text{a}\text{p}\text{p}}$ is a unitless parameter of the apparent kurtosis coefficient. MD and MK are the averages of ${\text{D}}_{\text{a}\text{p}\text{p}}$ and ${\text{K}}_{\text{a}\text{p}\text{p}}$ among three distributed directions, respectively.

Two radiologists (Z.X., W.J.)with respectively 5 and 8 years of experience in genitourinary imaging diagnosis independently processed, interpreted, reviewed and assessed the images using a double-blinded method. The delineation of regions of interest (ROIs) within the lesion was facilitated through the utilization of the syngo.via Frontier MR Multiparametric Analysis (Siemens Healthcare, Erlangen, Germany). Within the framework of the multi-parametric maps layout, the T2W- and T1W-enhanced images were employed as foundational references to depict ROIs along the tumor edge of each layer. Subsequently, these delineated ROIs were duplicated to corresponding MK, MD, ADC, pre- and post-contrast T1 maps. with necessary manual adjustments performed across distinct parametric maps in instances where there existed disparities between ROIs and the lesions. Areas of necrotic, hemorrhaging, or cystic lesions visible to the naked eye were avoided as much as possible. The parameter values in the ROIs on the generated parameter maps were subsequently recorded. The two radiologists performed statistical analysis using the averages of the measurements. Differences between native and postcontrast T1 (T1diff) values were calculated and averaged for both radiologists as follows:

$$ \begin{array}{l}{\rm{T1diff}}\,{\rm{ = }}\,\frac{{{\rm{native}}{\rm{.T1(observer1)}}\,{\rm{ + }}\,{\rm{native}}{\rm{.T1(observer2)}}}}{{\rm{2}}}\\\,\,\,\,\,\,\,\,\,\,\,\,\,\,\,\,\,\,\, - \frac{{{\rm{postcontrast}}{\rm{.T1(observer1)}}\,{\rm{ + }}\,{\rm{postcontrast}}{\rm{.T1(observer2)}}}}{{\rm{2}}}\end{array} $$

### Statistical analyses

SPSS 21.0 statistical software (IBM Corp., Armonk, NY, USA) and MedCalc statistical software (MedCalc Software, Mariakerke, Belgium) were used for the statistical analyses. The Kolmogorov-Smirnov test was performed to calculate data distributions. Quantitative data are expressed as means ± standard deviations for normally distributed data and medians (Q1, Q3) for skewed variables. Independent-sample t-tests and Mann-Whitney U tests were used to compare differences between groups. Receiver operating characteristic (ROC) curves were performed for all statistically significant variables, and the areas under the ROC curves (AUROCs) was calculated to to assess the differential diagnostic efficiency of each parameter. The optimal threshold value, as well as sensitivity and specificity were obtained. The DeLong test was applied to compare the AUROC of each parameter. Interobserver agreement was determined using inter-class correlation coefficients (ICCs). An ICC ≥ 0.75 was considered good agreement. A two-tailed *p* < 0.05 was considered statistically significant.

## Results

### Clinical characteristics

The characteristics of the 157 patients included in the study are summarized in Table 2.

**Table 2:** Patient characteristics

Characteristics	CSCC (*n* = 125)	CAC (*n* = 32)
Mean age, years (range)	50.4(27–77)	47.8(30–72)
Menstrual status, n		
Premenopausal	54	13
Postmenopausal	71	19
Tumor grade, n		
Low-grade (G1 + G2)	85	18
High-grade (G3)	40	14
FIGO stage, n		
IB	35	8
IIA	41	13
IIB	19	4
III	26	6
IV	4	1

### Interobserver agreement

All interobserver agreements for the measured parameters of cervical cancer were good or excellent. The ICCs were 0.813 (95% confidence interval [CI], 0.544–0.930) for native T1; 0.779 (95% CI, 0.476–0.971) for postcontrast T1; 0.765 (95% CI, 0.448–0.911) for MK; 0.858 (95% CI, 0.642–0.948) for MD; and 0.865 (95% CI, 0.656–0.951) for ADC.

### Differences in parameters

**Fig. 2:**
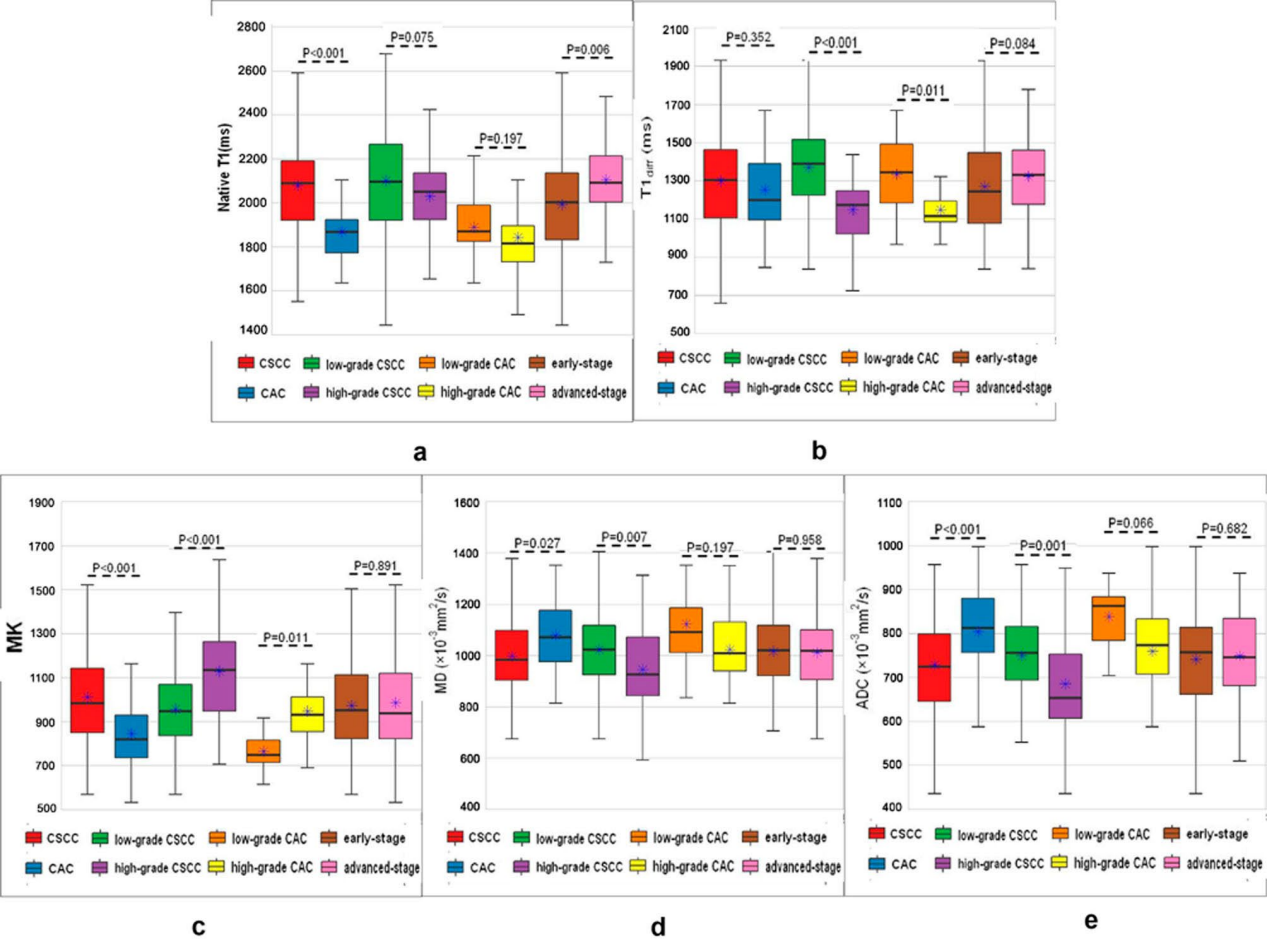
Boxplots of (**a**) native T1 values for all groups; (**b**) T1diff values for all groups; (**c**) MK values for all groups; (**d**) MD values for all groups; (**e**) ADC values for all groups

**Fig. 3:**
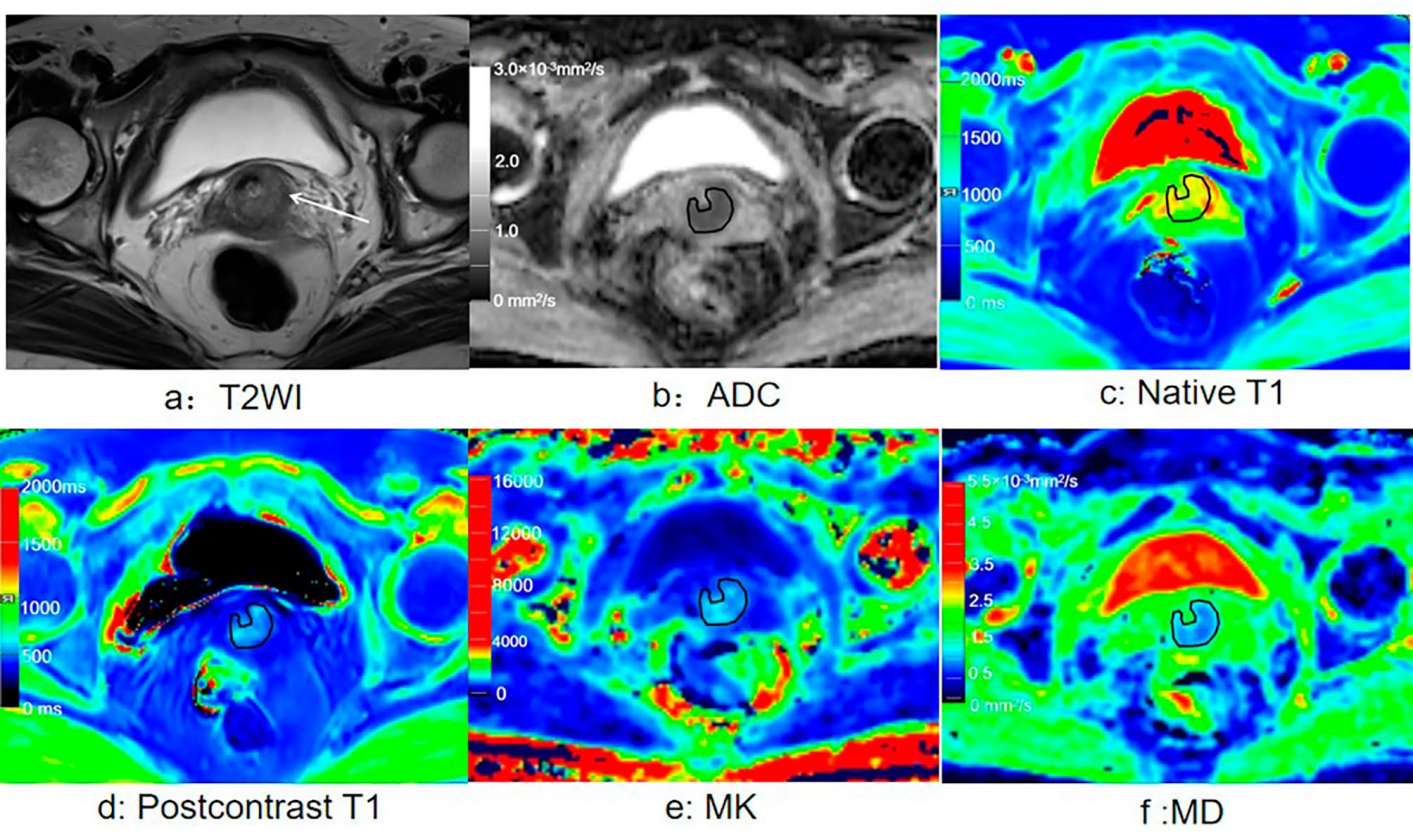
cervical squamous cell carcinoma (stage IB, high-grade). (**a**) Axial T2-weighted image shows a mass with a slight high-intensity signal in the cervix. (**b**) The mass showed low intensity on the ADC map. ADC value = 0.68 × 10 − 3 mm2/s. C) Native T1 map. d) Postcontrast T1 map. e) MK map. f) MD map

**Fig. 4:**
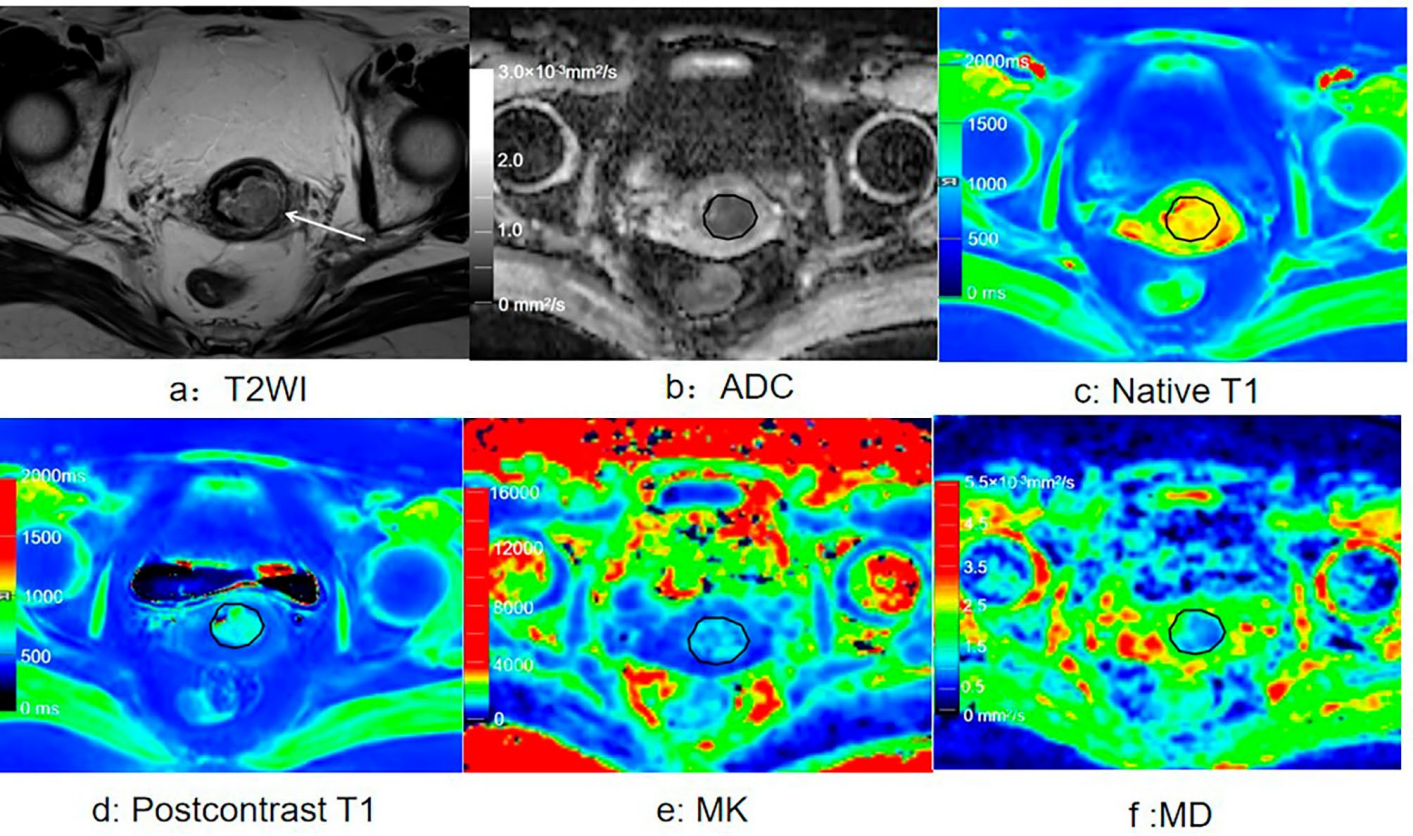
cervical adenocarcinoma (stage IB, low-grade). (**a**) Axial T2-weighted image shows a mass with a slight high-intensity signal in the cervix. (**b**) The mass showed low intensity on the ADC map. (**c**) Native T1 map. (**d**) Postcontrast T1 map. (**e**) MK map. (**f**) MD map

For the CSCC group, the native T1 and MK values were significantly higher, and the MD and ADC were significantly lower than those in the CAC group (all *p* < 0.05). For the high-grade CSCC group, T1_diff_, MD, and ADC were significantly lower (all *p* < 0.05), and MK values were significantly higher than those in the low-grade CSCC group (all *p* < 0.05). For the high grade CAC group,T1_diff_ was significantly lower, and MK was significantly higher than those in the low-grade CAC group (both *p* < 0.05). Although native T1 was significantly higher in the advanced-stage group than in the early-stage group (*p* < 0.05), the T1_diff_, MK, MD, and ADC values did not differ significantly between the two groups (all *p* > 0.05; Fig. 2; Table 3). Figure 3 shows representative images from a patient with stage IB, high-grade cervical squamous cell carcinoma. Images from a patient with stage IB, low-grade cervical adenocarcinoma are presented in Fig. 4.

**Table 3:** T1- and DKI- derived parameters in differentiating tumor subtype,grade and stage of cervical cancer

Groups	Native T1 (ms)	T1_diff_ (ms)	MK	MD (×10^− 3^ mm^2^/s)	ADC (×10^− 3^ mm^2^/s)
CSCC	2078.2 ± 238.6	1299.4 ± 264.6	0.98 [0.85, 1.15]	1.00 ± 0.15	0.73 ± 0.11
CAC	1867.7 [1752.2, 1946.4]	1252.2 ± 214.7	0.84 ± 0.17	1.07 [0.97, 1.18]	0.81 [0.75, 0.88]
*p*	< 0.001*	0.352	< 0.001*	0.027*	< 0.001*
High-grade CSCC	2029.7 ± 176.4	1148.2 ± 211.0	1.13 ± 0.24	0.94 ± 0.16	0.69 ± 0.12
Low-grade CSCC	2101.1 ± 260.7	1370.6 ± 258.1	0.96 ± 0.19	1.02 ± 0.14	0.75 ± 0.09
*p*	0.075	< 0.001*	< 0.001*	0.007*	0.001*
High-grade CAC	1815.7 [1679.4, 1897.5]	1147.8 ± 184.3	0.95 ± 0.17	1.02 ± 0.21	0.76 ± 0.16
Low-grade CAC	1889.7 ± 149.0	1333.4 ± 205.2	0.76 ± 0.12	1.09 [1.00, 1.19]	0.84 ± 0.07
*p*	0.197	0.011*	0.001*	0.197	0.066
Early stage CC	1992.5 ± 248.4	1242.5 [1071.8, 1450.0]	0.95 [0.82, 1.11]	1.02 [0.92, 1.12]	0.74 ± 0.11
Advanced stage CC	2105.3 ± 240.7	1322.7 ± 246.4	0.94 [0.82, 1.13]	1.01 ± 0.15	0.75 ± 0.11
*p*	0.006*	0.084	0.891	0.958	0.682

Abbreviations: DKI, diffusion kurtosis imaging; CSCC, cervical squamous cell carcinoma; CAC, cervical adenocarcinoma; MK, kurtosis along the axial direction; MD, mean diffusivity; ADC, apparent diffusion coefficient

*p < 0.05

### Area under the ROC (AUROC) curve analysis

**Fig. 5:**
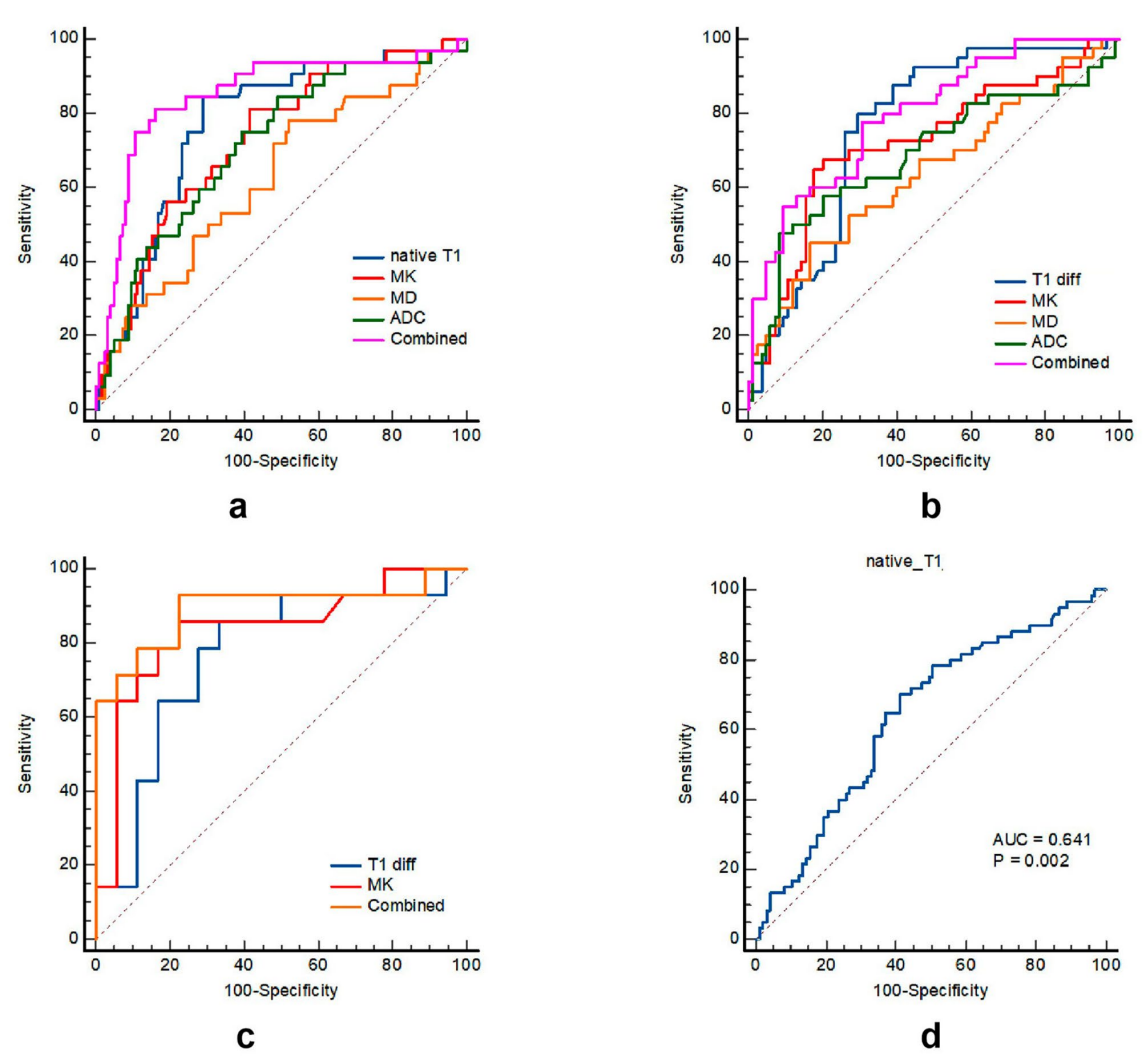
(**a**) ROC curve of all parameters for distinguishing CSCC and CAC. (**b**) ROC curve of all parameters for distinguishing high-grade CSCC and low-grade CSCC. (**c**) ROC curve of all parameters for distinguishing high-grade and low-grade CAC. (**d**) ROC curve of all parameters for distinguishing early-stage and advanced-stage cervical cancer

The order of the AUROCs for distinguishing CSCC from CAC was native T1 (0.772) > MK (0.731) > ADC (0.715) > MD (0.627), and only the AUROCs of native T1 and MD were statistically significantly different according to DeLong’s test (*p* = 0.045). Combined T1 mapping and DKI parameters could increase the AUC to 0.850 in distinguishing CSCC from CAC. The order of the AUROCs for distinguishing high-grade CSCC from low-grade CSCC was T1_diff_ (0.762) > MK (0.721) > ADC (0.689) > MD (0.640); however, these differences were not statistically significant (all *p* > 0.05). Combined T1 mapping and DKI parameters could increase the AUC to 0.796 in distinguishing high-grade CSCC from low-grade CSCC. For distinguishing high-grade and low-grade CAC, the AUROC of MK (0.835) was higher, but not statistically significant, than that of T1_diff_ (0.766, *p* = 0.550). Combined T1 mapping and DKI parameters could increase the AUC to 0.893 in distinguishing high-grade and low-grade CAC. For distinguishing advanced-stage from early-stage cervical cancer, only the AUROC of native T1 was significant (AUROC = 0.641, *p* = 0.002; Fig. 5; Table 4).

**Table 4:** Diagnostic performance of T1 mapping- and DKI-derived parameters

Category	Threshold	AUC (95%CI)	*p*-value	Sensitivity	Specificity
**CSCC vs. CAC**					
Native T1 (ms)	2002.9	0.772 (0.698–0.835)	< 0.001*	84.4%	71.2%
T1_diff_ (ms)	1203.0	0.558 (0.447–0.637)	0.288	53.1%	66.4%
MK	0.94	0.731 (0.655–0.799)	< 0.001*	88.2%	58.4%
MD (×10^− 3^ mm^2^/s)	0.97	0.627 (0.546–0.703)	0.027*	78.1%	48.0%
ADC (×10^− 3^ mm^2^/s)	0.76	0.715 (0.638–0.784)	< 0.001*	75.0%	60.8%
Combined		0.850(0.784–0.901)	< 0.001*	81.25%	84.0%
**CSCC grade: high vs. low**					
Native T1 (ms)	2157.5	0.592 (0.500–0.679)	0.071	85.0%	38.8%
T1_diff_ (ms)	1267.3	0.762 (0.677–0.833)	< 0.001*	80.0%	70.6%
MK	1.09	0.721 (0.634–0.797)	< 0.001*	67.5%	80.0%
MD (×10^− 3^ mm^2^/s)	0.90	0.640 (0.549–0.724)	0.012*	45.0%	83.5%
ADC (×10^− 3^ mm^2^/s)	0.64	0.689 (0.600-0.768)	0.001*	47.5%	91.8%
Combined		0.796(0.714–0.862)	0.001*	77.5%	69.4%
**CAC grade: high vs. low**					
Native T1 (ms)	1810.8	0.635 (0.447–0.797)	0.204	50.0%	83.3%
T1_diff_ (ms)	1224.4	0.766 (0.583–0.897)	0.003*	85.7%	66.7%
MK	0.82	0.835 (0.662–0.942)	< 0.001*	85.7%	77.8%
MD (×10^− 3^ mm^2^/s)	1.08	0.635 (0.447–0.797)	0.195	71.4%	61.1%
ADC (×10^− 3^ mm^2^/s)	0.85	0.694 (0.507–0.844)	0.060	85.7%	55.6%
Combined		0.893(0.732–0.974)		92.86%	77.78%
**Stage: advanced vs. early**					
Native T1 (ms)	2028.7	0.641 (0.561–0.716)	0.002*	70.0%	58.8%
T1_diff_ (ms)	1142.1	0.582 (0.501–0.660)	0.076	81.7%	40.2%
MK	0.86	0.507 (0.426–0.587)	0.893	60.0%	30.9%
MD (×10^− 3^ mm^2^/s)	0.82	0.502 (0.422–0.583)	0.958	5.0%	86.6%
ADC (×10^− 3^ mm^2^/s)	0.88	0.512 (0.431–0.593)	0.803	16.7%	92.8%

DKI, diffusion kurtosis imaging; AUC, area under the curve; CSCC, cervical squamous cell carcinoma; CAC, cervical adenocarcinoma; MK, kurtosis along the axial direction; MD, mean diffusivity; ADC, apparent diffusion coefficient

*p < 0.05

## Discussion

We conducted an initial investigation of the feasibility of quantitative T1 mapping for preoperatively assessing cervical cancer and compared it with the DKI results. The results showed that T1 mapping was deemed feasible for assessing cervical cancer. Compared with DKI-derived parameters, quantitative native T1 better differentiated tumor type and stage, and T1diff exhibited comparable discriminative value for tumor grade.

We found that native T1, MK, MD, and ADC were all valuable for identifying the cervical cancer pathological subtypes, CSCC and CAC. Native T1 achieved the highest diagnostic efficacy, although only the difference in the AUROC between MD and native T1 was statistically significant. In T1 mapping, the native T1 of tissues provides a quantitative analysis of the changes in internal tissue composition [15]. Native T1 is associated with several factors, including concentrations of proteins and polypeptides, water content of the tissue, and water binding status. Relatively small variations of native T1 in biological tissue can reflect pathological changes [11]. Meng et al. reported that the extracellular macromolecules correlated with cell necrosis were more abundant in malignant breast tumors, which may have contributed to a longer T1 relaxation time [24]. In this study, the native T1 values were higher in the CSCC group than in the CAC group, possibly because CSCC are more prone to severe tissue micronecrosis. DKI is an extension of DWI for detecting non-Gaussian distributions of water molecules in living tissues [25]. Unlike T1 mapping, DKI primarily reflects the degree of diffusion restriction and microstructure complexity [26]. CSCC has higher MK and lower MD and ADC values than does CAC, likely because of the different pathological characteristics of these cancers. CSCC originates from the cervical squamous epithelium and has a more compact cell distribution and smaller extracellular space than does CAC, thus exhibiting higher restrictions to water diffusion within the tumor tissue. Furthermore, tissue necrosis results in a more complex microstructure in CSCC tissue, causing a more pronounced deviation in water molecule diffusion from the Gaussian distribution. Conversely, cells with glandular duct-like structures result in a lower cell density and less tissue necrosis in CAC, and water molecule diffusion is closer to the Gaussian distribution, which may explain the reduced MK and increased MD and ADC values in CAC compared with those in CSCC.

Pathological grade is an independent prognostic factor for cervical cancer. High-grade cervical cancer indicates high tumor malignancy and a poor prognosis [27]. In our study, lower T1diff was associated with higher tumor grade, whereas native T1 was insufficiently sensitive to distinguish between high-grade and low-grade cervical cancer. T1diff, by being defined as the difference between postcontrast T1 and native T1, is another quantitative parameter of T1 mapping that eliminates some potentially confounding effects from postcontrast T1 measurements. T1diff measurements predominantly reflect changes in the extracellular space that are affected by tumor perfusion, vascular permeability, and tumor cell density. Adams et al. found that T1diff was closely associated with the pathological grade of renal clear cell carcinoma [14]. Wang et al. showed that T1 mapping after enhancement allows effectively identifying post-gamma knife radiosurgery recurrence and radiation necrosis in brain metastases [28]. A study on DCE-MRI showed that poorly differentiated cervical cancer had a significantly higher volume transfer coefficient (ktrans) and rate constant (Kep) than did well-to-moderately differentiated cervical cancer [29]. In this study, the T1diff was lower for high-grade than for low-grade cervical cancer, suggesting that the amount of contrast agent distributed in the extracellular space of high-grade tumors after 5 min of enhancement was lower in the high-grade group than in the low-grade group. This may be related to the increased amount of immature and highly permeable vessels in high-grade tumors due to neovascularization, through which the contrast agent enters the tissue interstitium and flows back into the microvasculature. High-grade CSCC also exhibited higher MK values and lower MD and ADC values than did low-grade CSCC. In high-grade CSCC tissues, increased cell density and nuclear-cytoplasmic ratios resulted in more restriction of intracellular and extracellular water diffusion, leading to lower MD and ADC values, while increased cell heterogeneity and structural complexity of the tissue resulted in higher MK values. These results are consistent with those of Hou et al. [30]. However, in the current study, MD and ADC had little significance in distinguishing the pathological grade of CAC, possibly owing to the sample size or the complexity of CAC pathological subtypes.

Our results showed that only native T1 could be used to distinguish early-stage from advanced-stage cervical cancer among T1 mapping and DKI parameters. Advanced-stage cervical cancer is characterized by a larger tumor size and large necrotic area, which may have contributed to a high native T1. Although necrotic areas visible to the naked eye were avoided during ROI selection, areas of micronecrosis can only be visualized microscopically and are therefore difficult to avoid. Regarding diagnostic efficacy, the AUROC of native T1 for distinguishing the tumor stage was 0.641, indicating only moderate efficacy. The high degree of native T1 overlap makes the results of native T1 alone unsuitable for predicting advanced-stage cervical cancer, and morphological images, such as high-resolution T2WI and enhanced T1WI, must be combined to comprehensively assess the tumor stage. Wang et al. reported that MD and ADC, but not MK, could distinguish histologic subtypes and FIGO stages [31]. However, no consensus exists regarding the value of DKI parameters for cervical cancer staging [28, ]. Therefore, large-sample, multicenter studies are required to further confirm the potential application of DKI in cervical cancer staging.

We compared the diagnostic performances of T1 mapping and DKI for predicting the subtype, grade, and stage of cervical cancer. ROC analysis revealed that native T1 had higher diagnostic efficacy for distinguishing the cervical cancer subtype and stage compared with those of DKI parameters, and T1diff had a diagnostic performance equivalent to MK for differentiating the pathological grade in CSCC and CAC. To date, few studies have compared T1 mapping and DKI in cervical cancer [20]. Previous studies that evaluated the pathological features of CSCC using multiparametric MRI found that the extravascular extracellular volume fraction (Ve) derived by DCE showed the best discriminative value for tumor grade [32]. Ve has a similar physical significance to T1diff but emphasizes different aspects. Ve is a quantitative parameter of DCE obtained from complex hemodynamic model calculations and is associated with tumor neovascular permeability, whereas T1diff is a composite of contrast agent distribution in the tissue. In clinical settings, DKI sequences require a longer scan time of 578 s, whereas T1 mapping sequences require a shorter scan time of only 30 s. As a commercially available sequence, T1 mapping is simpler and faster because it automatically generates direct measurements online, which may translate well into clinical practice. Additionally, T1 mapping before and after enhancement can provide multifaceted information on the tumor parenchyma and blood perfusion of cervical cancers, which can be later submitted to computer-aided evaluation systems. By combining T1 mapping with DKI, our results achieved a significant information gain in identifying pathological features of cervical cancer. Consequently, this gain led to an elevated AUROC. Multiparametric MRI facilitates a comprehensive delineation of the pathological characteristics to tumors from different dimensional perspectives. Multiparametric MRI combining T1mapping and DKI achieves high sensitivity and specificity, maximizing diagnostic accuracy.

This study had some limitations. First, there exists a discrepancy in the sample sizes between CSCC and CAC cohorts. Because CAC has a low incidence [2], the number of patients with CAC is small (*n* = 32). Nonetheless, it is noteworthy that the majority of previous studies on MRI functional imaging of cervical cancer have focused on squamous cell carcinoma of the cervix. In contrast, the presented study offers the largest number of CAC and different histologic grades with T1 mapping within the literature [16–20]. Second, rare histological subtypes, such as adenosquamous carcinoma and neuroendocrine tumors, were excluded, which may have caused selection bias. Third, multiple T1 mapping pulse sequences can be used to obtain T1 maps, and we used the VFA pulse sequence. Although this sequence is susceptible to B1 inhomogeneity in MR systems, we corrected this by applying the B1 map correction technique supplied by the manufacturer [33]. Future studies should further compare the potential of different T1 mapping sequences in assessing cervical cancer to find the most stable and accurate T1 mapping sequence. Fourth, considering the clinical utility and pharmacokinetic characteristics of contrast agents, contrast-enhanced T1 mapping sequence scans were performed 5 min after contrast injection. Further studies are needed to verify the feasibility of post-enhancement T1 mapping to assess cervical cancer at other time points.

## Conclusion

Compared with DKI-derived parameters, native T1 exhibits better efficacy for identifying cervical cancer subtype and stage, and T1diff exhibits comparable discriminative value for cervical cancer grade. T1 mapping is feasible as a noninvasive biomarker for preoperative assessment of cervical cancer and may translate well into clinical practice.

## Data Availability

The datasets used and/or analysed during the current study are available from the corresponding author on reasonable request.

## Abbreviations

MRI: Magnetic resonance imaging
DKI: Diffusion kurtosis imaging
DWI: Diffusion-weighted imaging
CSCC: Cervical squamous cell carcinoma
CAC: Cervical adenocarcinoma
VFA: Variable flip angle
T1W: T1-weighted
T2W: T2-weighted
VIBE: Volumetric interpolated breath-hold examination
RESOLVE: Readout-segmented echo-planar sequence
MK: Mean kurtosis
MD: Mean diffusivity
ADC: Apparent diffusion coefficient
ICC: Intra-group correlation coefficient
ROC: Receiver operating characteristic curve
AUC: Area under the curve
ROI: Regions of interest
FIGO: Federation International of Gynecology and Obstetrics
DCE: Dynamic contrast-enhanced
